# Supplementary material for: Sensitive red protein calcium indicators for imaging neural activity
Source: eLife. 2016 Mar 24;5:e12727. doi: 10.7554/eLife.12727 (PMC4846379; doi:10.7554/eLife.12727)
Supplement: Figure 2—source data 1. — Summary of red GECI biophysical properties, mean ± s.d., where indicated, for independently purified protein samples (Materials and methods). DOI: http://dx.doi.org/10.7554/eLife.12727.005 [file elife-12727-fig2-data1.docx]

| Sensor | Dynamic range (F_max_/F_min_) | K_d_  (nM) | Hill coefficient | k^off^  (s^-1^) | pK_a, apo_ | pK_a, sat_ | ε_apo_  (x1000)  (M^-1^cm^-1^) | ε_sat_  (x1000) |
| --- | --- | --- | --- | --- | --- | --- | --- | --- |
| R-GECO1 | 12.0 ± 0.4 | 337 ± 8 | 2.00 ± 0.04 | 7.0 | 8.7 | 6.4 | 5.34 | 54.9 |
| jRGECO1a | 11.6 ± 0.4 | 148 ± 2 | 1.90 ± 0.02 | 7.6 | 8.6 | 6.3 | 6.18 | 53.3 |
| RCaMP1h | 12.6 ± 0.6 | 1127 ± 35 | 2.20 ± 0.06 | 1.9 | 7.1 | 5.7 | 20.8 | 63.6 |
| jRCaMP1a | 3.2± 0.1 | 214 ± 10 | 0.86 ± 0.01 | 0.44 | 5.6 | 6.4 | 33.8 | 54.1 |
| jRCaMP1b | 7.2 ± 0.1 | 712 ± 9 | 1.60 ± 0.01 | 2.5 | 6.4 | 5.5 | 25.3 | 53.4 |
